# T Follicular Helper Cells and Regulatory B Cells Dynamics in Systemic Lupus Erythematosus

**DOI:** 10.1371/journal.pone.0088441

**Published:** 2014-02-14

**Authors:** Xue Yang, Ji Yang, Yiwei Chu, Yu Xue, Dandan Xuan, Shucong Zheng, Hejian Zou

**Affiliations:** 1 Division of Rheumatology, Huashan Hospital, Fudan University, Shanghai, China; 2 Institute of Rheumatology,Immunology and Allergy, Fudan University, Shanghai, China; 3 Institute of Molecular and Translational Medicine, Huashan Hospital, Fudan University; 4 Department of Dermatology, Zhongshan Hospital, Fudan University, Shanghai, China; 5 Department of Immunology, Shanghai Medical College, Fudan University, Shanghai, China; Pavillon Kirmisson, France

## Abstract

T follicular helper (Tfh) cells aid effector B cells, and augment autoimmunity, whereas the role of Tfh cells on regulatory B (Breg) cells in systemic lupus erythematosus (SLE) is not known. The aim of this study is to investigate the percentage of Breg cells in SLE, and the role of Tfh cells on Breg cells. First, we demonstrated the presence of Breg cells in SLE peripheral blood mononuclear cells and in involved skins. Both the percentage of circulating Breg cells and the ability to produce interleukin-10 (IL-10) were elevated in SLE patients. The percentage of Breg cells increased during SLE flares and decreased following disease remission. Second, Tfh cell expansion was not only related to autoantibody production but also correlated with the increased percentage of Breg cells. Third, in vitro studies revealed that Tfh cell-derived IL-21 could promote IL-10 production and Breg cell differentiation. In conclusions, these data imply that SLE flares may be linked to the expansion of Tfh cells and that Breg cells are increased in a regulatory feedback manner. Thus, SLE development may be associated with the complex regulation of Tfh cells and diverse B cell subsets.

## Introduction

Systemic lupus erythematosus (SLE) is an autoimmune disease that involves multiple organ systems [1]. The pathogenic mechanisms that cause lupus are unclear; however, the immune balance between regulatory T or B lymphocytes and effector T and B lymphocytes may be disturbed, contributing to the autoimmune injuries in SLE [2], [3], [4], [5].

Interleukin (IL)-10-producing regulatory B (Breg) cells have recently been identified. These cells, which represent 1∼3% of adult mouse spleen B cells, predominantly represent a subset of CD19^+^CD5^+^CD1d^high^ B cells and function to negatively regulate immune responses [3], [6], [7]. The absence or loss of Breg cells exacerbates disease symptoms in contact hypersensitivity, experimental autoimmune encephalomyelitis, chronic colitis, and collagen-induced arthritis models [8], [9], [10], [11]. IL-10 is a key cytokine produced by Breg cells, and diminished disease severity was observed following administration of IL-10 in the NZM2410 mouse model of lupus [12], whereas more severe disease occurred in both MRL/lpr mice on a IL-10 KO background and in Breg cell-deficient NZB/W mice [13], [14]. The finding that transfer of IL-10-secreting CD21^hi^CD23^hi^ B cells mitigates disease in MRL/lpr mice [15] further suggests that B cell-derived IL-10 limits disease activity. Although several studies showed that Breg cells were present in lupus-prone mice, including MRL/lpr and NZW mice [6], [13], [16], the dynamic change of Breg cells in SLE patients is not clear, and the mechanism of Breg cell differentiation in SLE patients is unknown.

T follicular helper (Tfh) cells, a subset of CD4^+^ T cells found in germinal centers (GCs), express high levels of C-X-C chemokine receptor type 5 (CXCR5), programmed death-1 (PD-1), and inducible costimulatory molecule (ICOS) [17], [18], [19]. Recently, expanded circulating Tfh cells were characterized as CD4^+^CXCR5^+^ICOS^high^PD-1^high^ in peripheral blood mononuclear cells (PBMCs) from SLE patients [20]. In addition, production of the CXCR5 ligand CXCL13 was also found to be elevated in SLE patients [21]. IL-21 is a key cytokine produced by Tfh cells [18], [19]. Our previous study demonstrated that the genotype and allele frequencies for copy number amplifications of IL-21 are significantly higher in SLE patients than in healthy controls [22]. Tfh cell-derived IL-21 is thought to drive the differentiation of B cells to produce antibodies, a process that serves as an important regulator of humoral immune responses [19], [23]. Recent studies showed that IL-21 is a pleiotropic cytokine, at least under specific circumstances, IL-21 can also exert anti-inflammatory actions due to its ability to inhibit dendritic cell maturation and stimulate IL-10 production in T cells [24], [25]. Our recent study proved that Tfh cell-derived IL-21 could promote the differentiation of Breg cells in lupus-prone MRL/lpr mice [16], however the relationship between Tfh and Breg cells in SLE patients is not known. Whether Tfh cell-derived IL-21 may also play a key role in the differentiation of Breg cells in SLE patients need be clarified.

Here, we provided evidence that Breg cells were present among PBMCs and involved skins in SLE patients. In detailed studies of Breg and Tfh cells from 30 SLE patients, we showed that Breg cells exhibited expansion rather than redistribution *in vivo*, and this expansion of Breg cells was related to disease activity. Further study demonstrated that expansion of Breg cells was related to Tfh cells in SLE. Tfh cell-derived IL-21 could promote IL-10 production and the differentiation of Breg cells. These data suggest that Tfh cell-derived IL-21 may induce the production of the anti-inflammatory cytokine IL-10 and result in expansion of Breg cells in SLE. Thus, the pathophysiology of SLE may be linked to a complex immune relationship between Tfh cells and diverse B subsets.

## Results

### Breg Cells are Expanded in SLE

**Figure 1:**
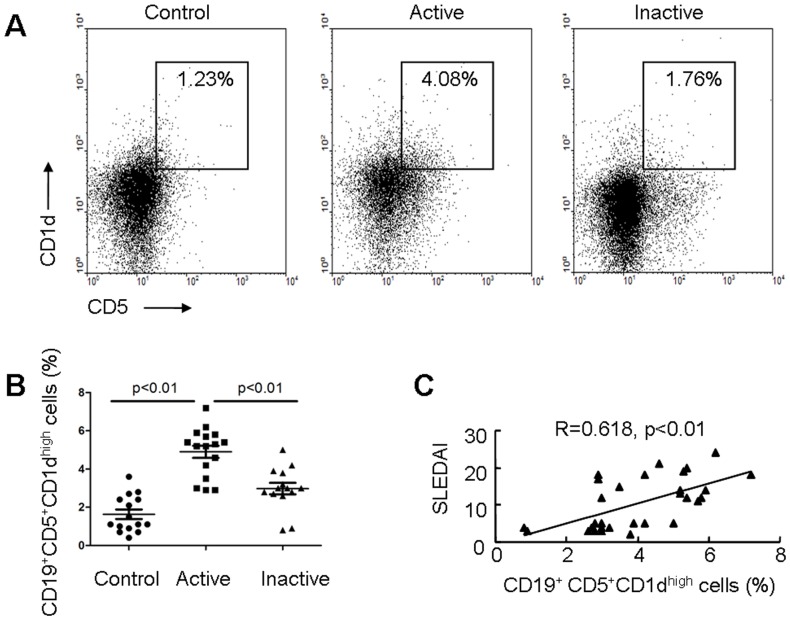
Expansion of circulating Breg cells in SLE patients. (A) Human PBMCs were labeled with lymphocyte-specific antibodies (CD19, CD5, and CD1d). The percentage of CD5^+^CD1d^high^ cells among a CD19 gate was determined by flow cytometry. (B) Results of flow cytometric analysis of Breg cells in patients with active SLE (n = 16), patients with inactive SLE (n = 14), and control subject (n = 15). (C) A positive correlation between the proportion of CD5^+^CD1d^high^ cells among CD19^+^ B cells and the clinical severity of the flare as scored using the SLEDAI (n = 30) was observed.

Since CD19^+^CD5^+^CD1d^high^ B cells with the capacity to negatively regulate immune responses have previously been named Breg cells [3], [6], [26], [27], we investigated these lymphocyte subgroups in 30 patients with SLE, including 16 active SLE patients and 14 inactive SLE patients. 15 healthy individuals were also included. The percentages of circulating CD19^+^CD5^+^CD1d^high^ B cells were measured by flow cytometry (Figure 1A). The percentage of circulating Breg cells was significantly increased in patients with active SLE (4.9±1.27%, n = 16) compared to inactive SLE (2.98±1.23%, n = 14) and healthy controls (1.63±0.99%, n = 15; Figure 1B). Furthermore, comparison of the percentage of Breg cells with the disease activity revealed a positive correlation between Breg cells and the SLEDAI value (R = 0.618, p<0.01, n = 30. Figure 1C). Although the absolute numbers of Breg cells were not significantly different between SLE patients and healthy controls, a positive correlation between absolute numbers of Breg cells and SLEDAI value was also detected (Figure S1A, B).

So far, other human Breg subsets have been described, namely CD24^high^CD27^high^ and CD24^high^CD38^high^ Breg cells in human and autoimmunity disease [28], [29]. Paul A Blair, et al showed that the percentage of CD24^high^CD38^high^ Breg cells was expanded in SLE, but displayed similar numbers of CD24^high^CD38^high^ Breg cells compared with healthy controls [29]. In our study, we observed that the percentages of CD19^+^CD24^+^CD38^+^ Breg cells were expanded in SLE patients than healthy controls, the absolute numbers were not significantly different between these two group (Figure S1C, D).

**Figure 2:**
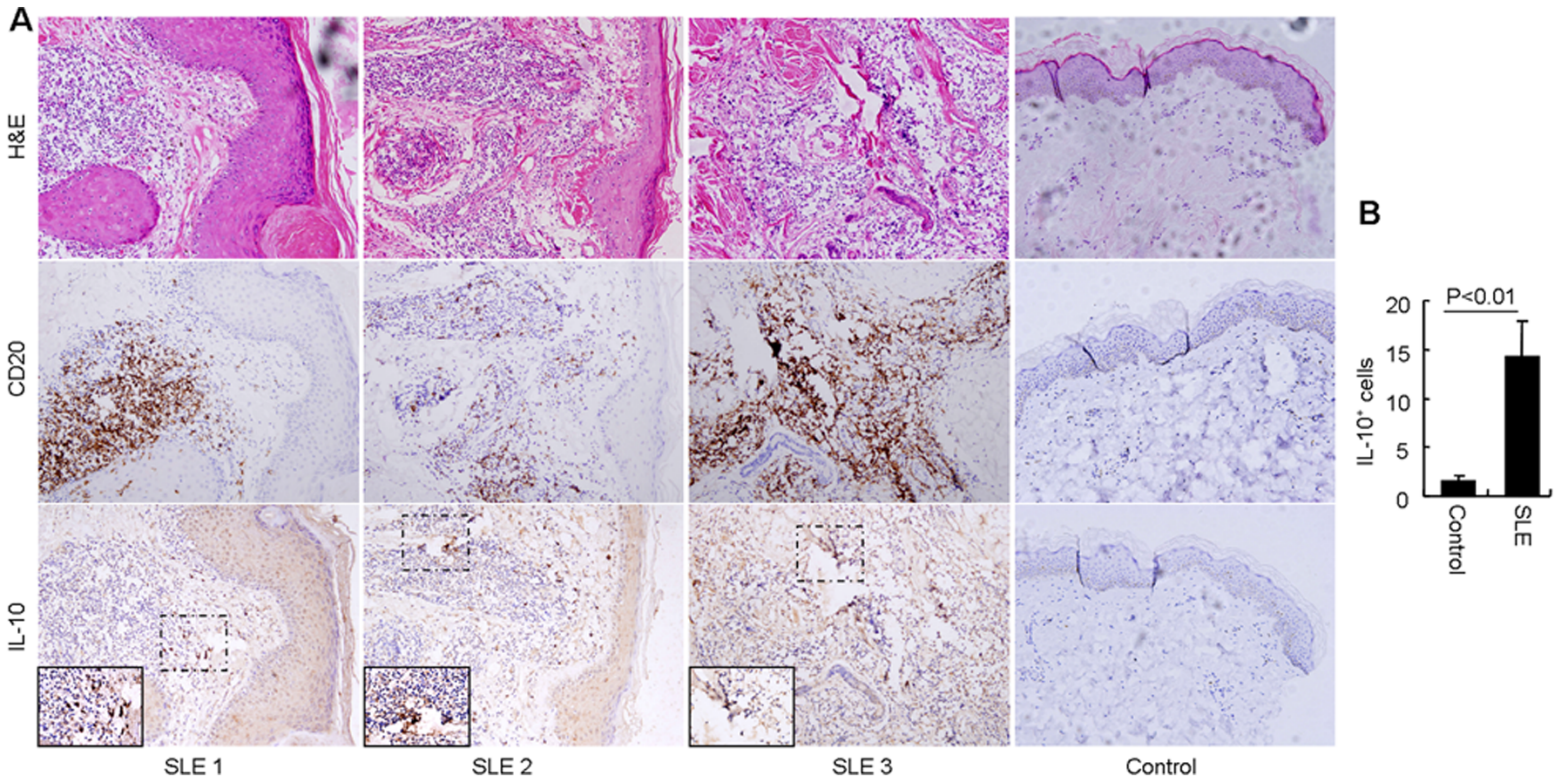
Infiltration of IL-10^+^ B cells in involved skin of SLE patients. (A) Consecutive levels obtained by serially section showed typical pathological changes of lupus (upper panel), lymphocytes infiltration confirmed by CD20 (middle panel) and IL-10 (lower panel) immunohistochemical staining (× 100 magnification). Further magnification of the black-bordered box shows typical IL-10^+^ lymphocytes (×400 magnification). (B) The counts of IL-10^+^ lymphocytes in skins were showed in right (SLE patients n = 10, healthy controls n = 4).

Breg cells with the capacity to produce IL-10 are also named B10 cells. To further investigate these B cell subgroups, we focused on the IL-10^+^ B cells in involved skins. Using immunohistochemistry, we analyzed skins from 10 SLE patients. The SLE patient samples exhibited typical pathological changes of lupus that the cornified layer exhibits patchy follicular plugging, lymphocytic interface dermatitis is seen, associated with basal layer keratinocytes degeneration, superficial and deep perivascular and periadnexal lymphocytic infiltration (Figure 2A, upper panel), including a large number of infiltrating CD20^+^ B cells (Figure 2A, middle panel). Examination of the IL-10^+^ cells in consecutive levels obtained by serially section confirmed that IL-10^+^ B cells were present (Figure 2A, lower panel, and Figure S2A). The presence of CD20^+^IL-10^+^ B cells in skin of SLE patient was further confirmed by immunofluorescence analysis (Figure S2B). These data indicate that Breg cells are present and expanded in SLE patients.

### Breg Cells Produce more IL-10 in SLE

**Figure 3:**
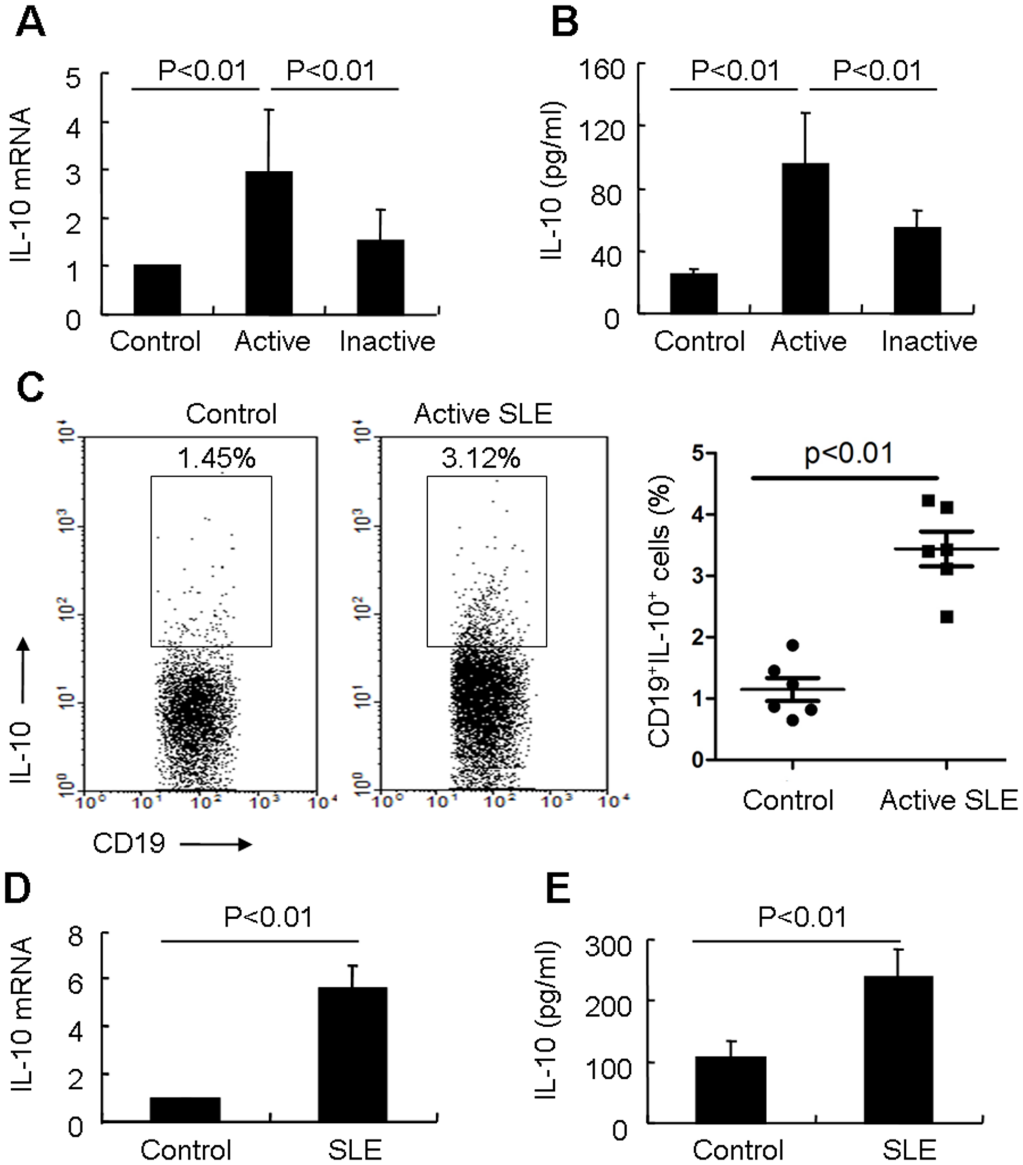
IL-10 production in Breg cells of SLE patients. (A) Real-time RT-PCR analysis of IL-10 mRNA expression in PBMCs from patients with active SLE (n = 16), patients with inactive SLE (n = 14), and control subject (n = 15). (B) Serum levels of IL-10 were detected in patients with active SLE (n = 16), patients with inactive SLE (n = 14), and control subject (n = 15) by ELISA. (C) PBMCs were isolated and stimulated with LPS for 24 hours and PIB for the final 5 hours. CD19^+^IL-10^+^ cells were detected by flow cytometry analysis in a CD19 gate (left). Results of flow cytometric analysis of CD19^+^IL-10^+^ cells (right, n = 6 for each group). (D) Sorted CD19^+^CD5^+^CD1d^high^ B cells from SLE patients and healthy controls were stimulated with LPS for 24 hours and PIB for the last 5 hours. IL-10 mRNA expression was detected by real-time RT-PCR. Results shown are representative of at least three independent experiments. (E) Sorted CD19^+^CD5^+^CD1d^high^ B cells from SLE patients and healthy controls were stimulated with LPS for 24 hours and PI for the last 5 hours. IL-10 in supernatants was detected by ELISA. Results shown are representative of at least three independent experiments.

IL-10 is a key cytokine produced by Breg cells [3], [9], our data showed that IL-10 mRNA was expressed at higher levels in PBMCs from active SLE patients than that in inactive SLE patients and healthy controls (Figure 3A). Furthermore, we confirmed that the secretion of IL-10 in sera from active SLE patients was significantly higher than that in sera from inactive patients and healthy controls (Figure 3B). Although our results implied that circulating Breg cells were expanded during the SLE disease process, multiple cell types can produce IL-10 [30], thus we further analyzed whether Breg cells in SLE patients have more ability of IL-10 production. We first proved that CD19^+^IL-10^+^ B cells were present in PBMCs of SLE patients by fluorescence microscopy (Figure S3A). Further study showed that the percentage of circulating CD19^+^IL-10^+^ B cells was expanded in patients with active SLE (3.44±0.69%, n = 6) than that of healthy controls (1.15±0.45%, n = 6, Figure 3C), while the absolute numbers of CD19^+^IL-10^+^ B cells were not significantly different between SLE patients and healthy controls (Figure S3B). Further examination revealed that IL-10 mRNA expression and protein secretion in sorted CD19^+^CD5^+^CD1d^high^ Breg cells from SLE patient was higher than that in sorted Breg cells from healthy control (Figure 3D, E). All together, these data suggest that Breg cells form SLE patients have more potential to produce IL-10.

### Tfh Cells are Related to the Expansion of Breg Cells in SLE

**Figure 4:**
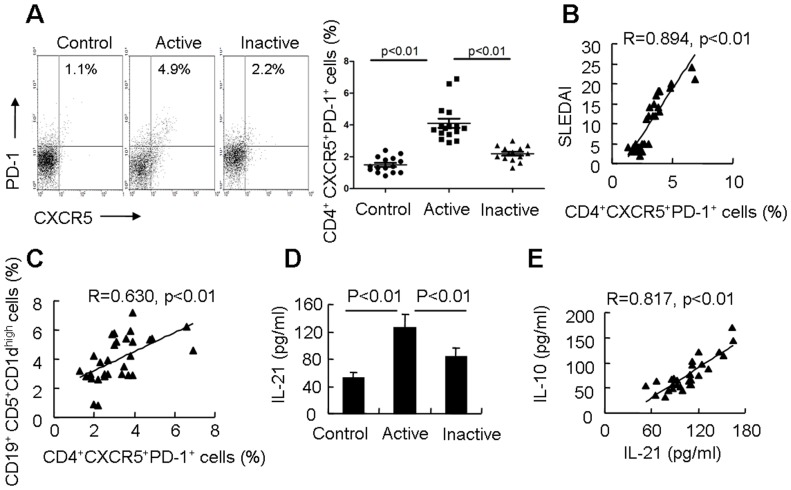
Tfh cells are associated with expansion of Breg cells in SLE patients. (A) Human PBMCs were labeled with lymphocyte-specific antibodies (CD4, CXCR5 and PD-1). The percentage of CXCR5^+^PD-1^+^ cells among CD4^+^ T cells was analyzed by flow cytometry (left). Results of flow cytometric analysis of Tfh cells (right) in patients with active SLE (n = 16), patients with inactive SLE (n = 14), and control subject (n = 15). (B) A positive correlation between the proportion of CXCR5^+^PD-1^+^ cells among CD4^+^ T cells and the clinical severity of the flare as scored using the SLEDAI (n = 30) was noted. (C) A positive correlation between the proportion of CD4^+^CXCR5^+^PD-1^+^ T cells and CD19^+^CD5^+^CD1d^high^ B cells in PBMCs from SLE patients (n = 30) was observed. (D) Serum IL-21 levels were detected in patients with active SLE (n = 16), patients with inactive SLE (n = 14), and control subject (n = 15) by ELISA. (E) A positive correlation between serum IL-10 levels and IL-21 levels in SLE patients (n = 30) was observed.

Tfh cells, a subset of CD4^+^ T cells, are well described as CXCR5^+^PD-1^+^ and mainly produce IL-21 [17], [18], [31]. We first detected CXCR5^+^PD-1^+^ T cells in PBMCs of SLE patients by fluorescence microscopy (Figure S4A). The percentage of circulating Tfh cells in PBMCs of patients with SLE was determined by flow cytometry. Our data showed that the percentage of Tfh cells was significantly increased in active SLE patients (4.11±1.17%, n = 16) compared with inactive SLE patients (2.19±0.47%, p<0.01, n = 14) and healthy controls (1.49±0.48%, p<0.01, n = 15; Figure 4A). Furthermore, we also found a positive correlation between percentage of Tfh cells and the SLEDAI (R = 0.894, p<0.01, n = 30. Figure 4B). Interesting, a strong positive correlation between the proportion of Tfh cells and Breg cells in SLE patients was also found (R = 0.630, p<0.01, n = 30. Figure 4C). However the absolute numbers of Tfh cells were not significantly different between SLE patients and healthy controls (Figure S4B).

Our data confirmed that the secretion of IL-21 in sera from active SLE patients was significantly higher than that in sera from inactive SLE patients and healthy controls (Figure 4D). The ds-DNA titers in sera of SLE patients were checked by ELISA, and a positive correlation between serum IL-21 levels and ds-DNA titers in sera of SLE patients was detected (data not shown). In addition, a strong positive correlation between the IL-21 and IL-10 serum levels in SLE patients was observed (R = 0.817, p<0.01, n = 30. Figure 4E). These data indicate that Tfh cells are expanded in SLE patients and that Tfh cells may be involved in the expansion of Breg cells in SLE patients.

### Tfh Cell-derived IL-21 Promotes IL-10 Production during the Differentiation of Breg Cells

**Figure 5:**
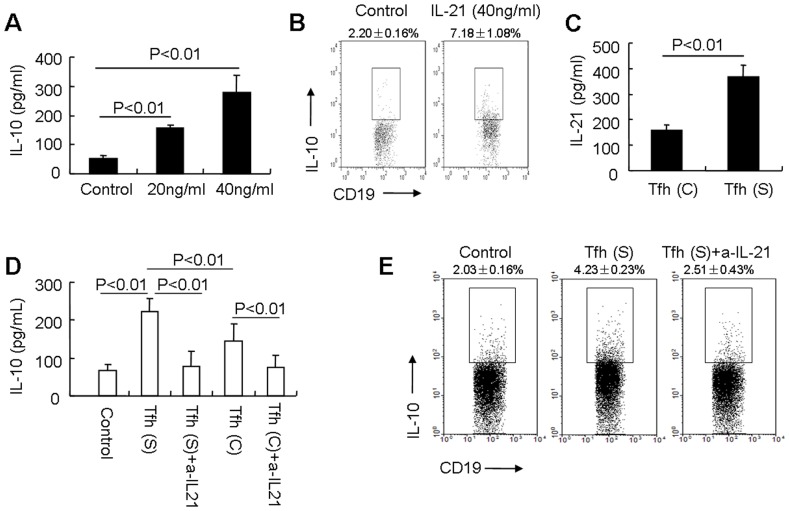
Tfh cell-derived IL-21 promotes IL-10 production during the differentiation of Breg cells. (A) Sorted B cells (CD43 depletion) from healthy controls were cultured in the presence of LPS and the indicated concentrations of IL-21 for 48 hours followed by stimulation with PI for the final 5 hours (Control: LPS+PIB). IL-10 in supernatants was detected by ELISA. Results shown are representative of at least three independent experiments. (B) Sorted B cells (CD43 depletion) from healthy controls were cultured in the presence of LPS with or without 40 ng/ml IL-21 for 48 hours followed by stimulation with PIB for the last 5 hours (Control: LPS+PIB). IL-10^+^ cells were analyzed in a CD19 gate by flow cytometry. Results shown are representative of at least three independent experiments. (C) Sorted CD4^+^CXCR5^+^PD-1^+^ T cells were cultured with anti-CD3 and anti-CD28 for 48 hours, IL-21 was detected in the supernatants by ELISA. Results shown are representative of at least three independent experiments (C: healthy controls; S: SLE patients). (D) Sorted B cells (CD43 depletion) were stimulated for 48 hours with LPS plus supernatants from cultures of Tfh cells of SLE patients with or without neutralization of IL-21. The cells were also stimulated with PI for the last 5 hours. Levels of IL-10 in the supernatants were determined by ELISA (Control: LPS+PI; Tfh (S): LPS+supernatants from Tfh cells of SLE patients+PI; Tfh (C): LPS+supernatants from Tfh cells of healthy controls+PI). Results shown are representative of at least three independent experiments. (E) Sorted B cells (CD43 depletion) were cultured for 48 hours in the presence of LPS plus supernatants from Tfh cells of SLE patients. These cells were cultured with or without neutralization of IL-21 and were stimulated with PIB for the last 5 hours (Control: LPS+PIB; Tfh (S): LPS+supernatants from Tfh cells of SLE patients+PIB). IL-10^+^ cells were analyzed by flow cytometry with a CD19 gate. Results shown are representative of at least three independent experiments.

Our data showed that IL-21 could promote IL-10 secretion during the differentiation of Breg cells (Figure 5A). Furthermore, IL-21 in concert with LPS and PIB promoted the differentiation of CD19^+^IL-10^+^ B cells (Figure 5B), which is consistent with the recently Nature published results that IL-21 is important for Breg cell expansion and IL-10 production [32]. To determine whether Tfh cell-derived IL-21 from SLE patients induces IL-10 production during the differentiation of Breg cells, CD4^+^CXCR5^+^PD-1^+^ Tfh cells were first sorted from PBMCs of active SLE patients and healthy controls and stimulated with anti-CD3 and anti-CD28 for 48 hours. IL-21 secretion in the supernatants of the cultured Tfh cells from active SLE patients was significantly higher than that from Tfh cells from healthy controls (p<0.01. Figure 5C). We next examined the effects of the supernatants from cultured Tfh cells on IL-10 production during the differentiation of Breg cells. Supernatants from Tfh cells of SLE patients promoted IL-10 production during Breg cell differentiation (Figure 5D). More notably, neutralization of IL-21 in the culture medium inhibited IL-10 secretion by Breg cells (Figure 5D). Furthermore, the supernatants from Tfh cells of SLE patients promoted CD19^+^IL-10^+^ cell differentiation, and neutralization of IL-21 in the culture medium inhibited this differentiation of Breg cells (Figure 5E). The results were further confirmed that the supernatants from Tfh cells of SLE patients promoted part of CD20^+^CD27^−^ naïve B cells differentiate into IL-10^+^ cells in the presence of LPS plus PIB (Figure S5). These data suggest that Breg cells are responsive to the stimulation by IL-21 that is produced by SLE patient-derived Tfh cells.

Taken together, these data confirmed that Tfh cells and Breg cells are expanded in SLE patients and that these cell subsets are correlated in these patients as well. Tfh cell-derived IL-21 may be involved in Breg cell expansion and IL-10 overproduction in SLE patients.

## Discussion

The ability of B cells to negatively regulate cellular immune responses and inflammation has been described previously [7]. Most recently, CD19^+^CD5^+^CD1d^high^ B cells with the capacity to produce IL-10 have been named Breg cells (B10) in mice [3], [6], [7]. Remarkably, Breg cells are potent negative regulators of inflammation and autoimmunity in mouse models of disease *in vivo* [10], [11], [33]. Recently, IL-10-producing CD1d^high^, or CD5^+^IL-10^+^ Breg cells were identified in human [26], [27], [34], however little is known the dynamic changes of Breg cells in active or inactive SLE patient.

The balance between Breg cell negative regulation and B-cell positive contributions to immune responses are likely to vary in different diseases as well as during the course of disease. Breg cell numbers increase during some autoimmunity animal models like NZB/W mice [6], [13], our recent data proved that Breg cells were expanded in MRL/lpr mice [16]. Here, we demonstrated that the percentage of peripheral blood CD19^+^CD5^+^CD1d^high^ Breg cells was significantly increased in active SLE patients and was positively correlated with disease activity, Breg cells decreased during disease relief. Breg cells produced more IL-10 in active SLE patients than healthy control. In addition, more IL-10^+^ B cells were detected in involved skin of SLE patients when compared with controls. In addition, the percentage of CD19^+^CD24^+^CD38^+^ Breg cells was also expanded in SLE patients than heanlty control, which was consistent with previous results [29]. The absolute numbers of CD19^+^CD5^+^CD1d^high^ cells, CD19^+^CD24^+^CD38^+^ cells, and CD19^+^IL-10^+^ cells increased but not significantly in SLE patients when compared with healthy controls, which might be attributed to peripheral lymphopenia in SLE patients during flares.

The percentage of Breg cells was expanded in SLE patients and decreased following remission than in healthy controls, these data suggested that Breg cells are dynamic during the development of autoimmunity. Maintaining immunological balance involves the capacity of the immune system to upregulate immunosuppressive responses, which may limit deterioration by the autoimmune response. The upregulation of Breg cells in active SLE patients may reflect a regulatory feedback mechanism to restore cellular tolerance and ameliorate harmful autoimmune responses.

B10 cells were functionally identified by their ability to express cytoplasmic IL-10 after 5 hours of ex vivo stimulation, whereas progenitor B10 (B10pro) cells required 48 hours of in vitro stimulation before they acquired the ability to express IL-10. Recent study showed that the percentages of B10 cells in SLE patients were not significantly different from controls, but the percentages of B10+Bpro cells in SLE patients were significantly different from controls [28], these data implied that B cells in SLE have more potential to produce IL-10. In our study, modified methods were taken, the B cells were stimulated with LPS 24 hours and the last 5 hours of PIB stimulation, which was based on the previous reported methods [6]. Consistent with previous results [28], [35], our study confirmed that both IL-10 production and the percentage of CD19^+^IL-10^+^ B cells were increased in SLE patients; however, the reason behind this expansion of Breg cells in SLE was not addressed in the previous studies [28]. Our data showed that the absolute numbers of CD4^+^CXCR5^+^PD-1^+^ Tfh cells were not significantly increased in SLE patients than in healthy controls, however the percentage of CD4^+^CXCR5^+^PD-1^+^ Tfh cells were expanded in active SLE patients and that Tfh cell-derived IL-21 contributed to autoantibody production. Further analysis showed that the percentage of Tfh cells was positively related to disease activity in SLE, which suggested that Tfh cells may contribute to autoimmunity by helping B effector cells and inducing humoral immunity [19], [36]. Secondly, we unexpectedly identified a strong positive correlation between Tfh cells and Breg cells in SLE patients, suggesting that Tfh cells may contribute to the expansion of Breg cells in SLE. Our *in vitro* data further revealed that SLE patient Tfh cell-derived IL-21 in synergy with LPS and PI promoted IL-10 production and the differentiation of Breg cells. This finding was verified as treatment of these cultures with an IL-21-neutralizing antibody inhibited IL-10 production and the generation of CD19^+^IL-10^+^ cells. IL-21 is a pleiotropic cytokine, and at least under certain circumstances, IL-21 can stimulate anti-inflammatory IL-10 production in T and B cells [24], [25], [32], [37]. The generation of T and B subsets during autoimmune disease requires complex and reciprocal regulation; thus, micro-environmental cytokines or other factors may be involved in the development of pro-inflammatory or anti-inflammatory lymphocyte subsets. Our data suggest that Tfh cells facilitate immune homeostasis by increasing the number of regulatory B cells and the production of IL-10 via the stimulation of IL-21 in SLE patients.

All together, we define a novel role of Tfh cells in immune regulatory actions to promote production of the immunosuppressive cytokine IL-10, which extends the existing recognization that Tfh cells merely induce humoral responses and augment autoimmunity. Furthermore, IL-21 may serve as a potential upstream promoter for Breg cell differentiation and IL-10 production in SLE. These findings suggest that particular emphasis should be given to the regulatory function of Tfh cells and IL-21 in the treatment of SLE.

## Materials and Methods

### SLE Patients and Healthy Controls

This study was approved by the Ethical Committee of Huashan Hospital and Zhongshan Hospital, Fudan University (Shanghai, People’s Republic of China). Thirty consecutive adult patients (28 women and 2 men, mean age 37.6±12.3 years) with a diagnosis of SLE, based on the American College of Rheumatology 1997 revised criteria [38], were included in the study. All patients enrolled in the study after giving informed and written consent. All SLE patients were referred to the Division of Rheumatology, Huashan Hospital or to the Department of Dermatology, Zhongshan Hospital, Fudan University, Shanghai, China. Disease activity was assessed by the SLE Disease Activity Index (SLEDAI). One group comprised subjects with active SLE (SLEDAI ≥6, n = 16, mean age 35.9±12.0 years, 15 women and 1 man), while the second group comprised subjects with inactive SLE (SLEDAI <6, n = 14, mean age 39.6±12.7 years, 13 women and 1 man) [2]. The following treatment was provided for the SLE group: prednisone, hydroxychloroquine+prednisone, or hydroxychloroquine +prednisone+cyclophosphamide. For the control group, 15 age and sex matched healthy individuals (mean age 36.2±12.7 years; 14 women and 1 man) were enrolled after giving informed consent. The ages, sex, and treatments of the patients are presented in Table 1.

**Table 1:** Patient characteristics.

Sex	Age, y	Disease duration, y	Treatment	SLEDAI score
F	41	3	HCQ	3
F	20	2	HCQ+Pred 10 mg/d	4
F	43	3	HCQ+Pred 15 mg/d	5
F	61	16	Pred 15 mg/d	4
M	45	7	Pred 20 mg/d	3
F	21	2	Pred 12.5 mg/d	4
F	36	10	Pred 7.5 mg/d	2
F	34	1.5	Pred 20 mg/d	4
F	44	3	HCQ+Pred 12.5 mg/d	5
F	36	11	Pred 12.5 mg/d	5
F	52	14	Pred 15 mg/d	5
F	61	10	None	3
F	32	1	Pred 10 mg/d	3
F	29	3	HCQ+Pred 15 mg/d	5
F	20	0.8	HCQ+Pred 35 mg/d	17
F	21	1	HCQ+Pred 50 mg/d	24
F	45	0.3	HCQ+Pred 25 mg/d	11
F	56	20	Pred 25 mg/d	13
F	44	7	Pred 20 mg/d	19
F	28	10	Pred 10 mg/d	12
M	38	0.5	Pred 15 mg/d	12
F	34	0.4	Pred 20 mg/d	18
F	43	15	None	21
F	23	2.5	Pred 20.25 mg/d	15
F	56	12	Pred 15 mg/d+CTX	18
F	27	0.5	HCQ+Pred 30 mg/d	12
F	28	0.9	Pred 15 mg/d	18
F	27	1	HCQ+Pred 50 mg/d	20
F	51	3	HCQ+Pred 15 mg/d	14
F	33	2	Pred 20 mg/d	14

HCQ = hydroxychloroquine; Pred = prednisone; CTX = cyclophosphamide.

### B and T Cell Isolation, Culture Conditions, and Differentiation

Human B cells were purified from PBMCs of healthy donors (CD43 depletion) by negative selection following the manufacturer’s instructions (Miltenyi Biotec, Bergisch Gladbach, Germany). For the differentiation of Breg cells, purified B cells (2×10^6^ cells/ml) were cultured in 10 µg/ml lipopolysaccharide (LPS; Sigma-Aldrich, St. Louis, MO) for 24 or 48 hours and stimulated with PIB (50 ng/ml phorbol 12-myristate 13-acetate [PMA], Sigma-Aldrich; 500 ng/ml ionomycin [Sigma-Aldrich]; and 20 µg/ml brefeldin A [BFA], eBioscience, San Diego, CA) for the last 5 hours, as previously described [6], [28]. Where indicated, cultures were supplemented with indicated doses of IL-21 (PeproTech, RockyHill, NJ) and LPS for 48 hours, and stimulated with PIB for the last 5 hours. In experiments to detect IL-10 in culture supernatants, BFA was not added. For some experiments, CD20^+^CD27^−^ naïve B cells (eBioscience) were sorted from PBMCs of healthy donors by flow cytometry, and cultured in certain conditions.

To determine the effects of Tfh cell-derived IL-21 on the activation of Breg cells, CD4^+^CXCR5^+^PD-1^+^ Tfh cells from active SLE patients were first sorted by flow cytometry. The resultant Tfh cells (2×10^6^ cells/ml) were stimulated with 2 µg/ml plate-bound anti-CD3 and 2 µg/ml soluble anti-CD28 (eBioscience) for 48 hours. Supernatants were collected for later use. Purified B cells or naïve B cells (2×10^6^ cells/ml) from healthy donors were cultured with 10 µg/ml LPS (Sigma-Aldrich) in the presence or absence of 20% supernatants from the above-stimulated Tfh cells or 20 µg/ml anti-IL-21 neutralizing antibody (eBioscience) for 48 hours. Culture media with the same doses of anti-CD3 and anti-CD28 was used as a vehicle control. Cultures were stimulated with PIB for the last 5 hours. IL-10^+^ cells were analyzed by flow cytometry with a CD19 gate. In experiments to detect IL-10 in culture supernatants by enzyme-linked immunosorbent assay (ELISA), BFA was not added. For some experiments, CD19^+^CD5^+^CD1d^high^ Breg cells (4×10^5^ cells) were obtained via cell sorting from PBMCs of SLE patients and healthy controls and were then cultured in the presence of LPS for 24 hours and PIB for the last 5 hours for the detection of IL-10 mRNA expression. For detecting IL-10 in culture supernatants, BFA was not added.

### ELISA

Sera from SLE patients and healthy controls were collected and frozen at −80°C until needed. Concentrations of anti-double-stranded DNA (anti-dsDNA) were determined by ELISA (R&D, Minneapolis, MN). Serum levels of IL-21 and IL-10 in SLE patients were also detected by commercial ELISA (eBioscience). In some experiments, isolated B cells (5×10^5^ cells) were cultured and stimulated with PMA and ionomycin (PI, Sigma-Aldrich) for the last 5 hours. IL-10 was detected in the supernatants by ELISA (eBioscience). Sorted CD4^+^CXCR5^+^PD-1^+^ Tfh cells (5×10^5^ cells) were stimulated with 2 µg/ml plate-bound anti-CD3 and 2 µg/ml soluble anti-CD28 (eBioscience) for 48 hours. IL-21 in supernatants was detected by ELISA (eBioscience).

### Flow Cytometry

For detection of Tfh cells, human PBMCs were stained with Alexa Fluor 647-conjugated anti-CD4, Alexa Fluor 488-conjugated anti-CXCR5, and phycoerythrin (PE)-conjugated anti-PD-1 (all from BD Pharmingen, San Jose, CA). Cells were gated for CD4^+^ T cells first and then for CXCR5^+^PD-1^+^ Tfh cells. For detection of Breg cells, PBMCs were stained with PerCP/Cy5.5-conjugated anti-CD19, fluorescein isothiocyanate (FITC)-conjugated anti-CD5, and PE-conjugated anti-CD1d (eBioscience) for 15 minutes. CD5^+^CD1d^high^ cells were analyzed with a CD19^+^ gate.

For intracellular IL-10 staining, PBMCs were incubated for 24 hours with 10 µg/ml LPS and stimulated with PIB for the last 5 hours. Surface staining with PerCP/Cy5.5-conjugated CD19 or FITC-conjugated anti-CD5 was first performed for 15 min, and cells were re-suspended in Fixation/Permeabilization solution (Invitrogen). Intracellular staining of PE-conjugated anti-IL-10 was performed according to the manufacturer’s protocol (eBioscience). After staining, IL-10^+^ cells were analyzed with a CD19^+^ gate by flow cytometry. For some experiments, cells were stained with FITC-conjugated CD19 and PE-conjugated anti-IL-10 (eBioscience) and detected by immunofluorescence microscopy.

### Immunohistochemistry

Skin biopsies from 10 SLE patients were obtained after informed consent, 4 normal skin biopsies (Three skin biopsies were from healthy donors after informed consent, one tissue was obtained from orthopedic surgery after informed consent) were used as controls. Tissues were processed and embedded in paraffin using routine methods. Tissue blocks were serially sectioned to obtain consecutive levels. Sections were stained with hematoxylin and eosin, and immunohistochemistry with the following antibodies was performed as previously described [2]. Antibodies to CD20 and IL-10 (Abcam, Cambridge, MA) were used. Immunohistochemical staining was assessed by two independent pathologists without knowledge of patient characteristics. The positive cells in per surface were counted under ×400 magnification, and five randomly selected independent microscopic fields were counted for each sample to ensure that the data were representative and homogeneous.

### Analyses of Cytokine and Transcription Factor mRNA Expression

Total RNA was purified with the Trizol reagent (Invitrogen). cDNAs were synthesized using Primescript RT Master Mix Perfect Real-time Kit (TaKaRa, Tokyo, Japan), and mRNA expression was determined with the Bio-Rad iCycler 7500 Optical System (Bio-Rad, Richmond, CA) using a SYBR Premix EX Taq Real-time PCR Master Mix (TaKaRa). The 2^−ΔΔCt^ method was used to normalize transcription to β-actin and to calculate the fold induction relative to controls. The following primer pairs were used: Hum β-actin, forward ATCATGTTTGAGACCTTCAACA and reverse CATCTCTTGCTCGAAGTCCA and Hum IL-10, forward GAAGTGAAAACGAGACCAAGGT and reverse CTGCAAGTTAGATCCTCAGG.

### Statistical Analyses

Results were expressed as means ± standard deviation. The statistical significance was determined by analysis of variance for comparisons of multiple means followed by the Bonferroni post hoc test, or the Student’s *t*-test, and the Mann-Whitney U-test. Correlations were determined by Spearman’s ranking.

## Supporting Information

Figure S1 **The absolute numbers of Breg cells in SLE patients.** (A) The results of flow cytometric analysis of absolute numbers of CD19^+^CD5^+^CD1d^high^ cells in patients with SLE (n = 30) and healthy controls (n = 15). (B) A positive correlation between the absolute numbers of CD19^+^ CD5^+^CD1d^high^ cells and the clinical severity of the flare as scored using the SLEDAI (n = 30) was observed. (C) Human PBMCs were labeled with lymphocyte-specific antibodies (CD19, CD24, and CD38). The percentage of CD24^+^CD38^+^ cells among a CD19 gate was determined by flow cytometry (left). Results of flow cytometric analysis of percentage of CD24^+^CD38^+^ cells among a CD19 gate cells in patients with SLE and control subject (right, n = 7 for each group). (D) The results of flow cytometric analysis of absolute numbers of CD19^+^CD24^+^CD38^+^ cells in patients with SLE and healthy controls (n = 7 for each group).
(TIF)

Figure S2 **IL-10^+^ cells in skins of SLE patients.** (A) The skin tissues from SLE patient were serially sectioned to obtain consecutive levels. The sections were stained with antibodies to IL-10 and isotype control. (B) The skin tissues from SLE patient were stained with CD20 and IL-10, the CD20^+^IL-10^+^ cells were analyzed by immunofluorescence microscopy.
(TIF)

Figure S3 **IL-10^+^ cells in PBMCs of SLE patients.** (A) PBMCs were isolated and stimulated with LPS for 24 hours and PIB for the final 5 hours. The presence of CD19^+^IL-10^+^ cells in PBMCs from active SLE patients was detected by immunofluorescence microscopy. The arrow indicates typical positive cells. (B) CD19^+^IL-10^+^ cells were detected by flow cytometry analysis in a CD19 gate (n = 6 for each group).
(TIF)

Figure S4 **Tfh cells in PBMCs of SLE patients.** (A) The presence of CXCR5^+^PD-1^+^ cells in PBMCs of active SLE patients was detected by immunofluorescence microscopy. The arrow indicates the typical positive cells. (B) The results of flow cytometric analysis of absolute numbers of CD4^+^CXCR5^+^PD-1^+^ cells in patients with SLE (n = 30) and healthy controls (n = 15).
(TIF)

Figure S5 **Tfh cell-derived IL-21 promotes the differentiation of Breg cells.** Sorted CD20^+^CD27^−^ naïve B cells were cultured for 48 hours in the presence of LPS plus supernatants from Tfh cells of SLE patients. These cells were cultured with or without neutralization of IL-21 and were stimulated with PIB for the last 5 hours (Control: LPS+PIB; Tfh (S): LPS+supernatants from Tfh cells of SLE patients+PIB). IL-10^+^ cells among the sorted B cells were analyzed by flow cytometry. Results shown are representative of at least three independent experiments.
(TIF)
