# Supplementary figures and images for: T Follicular Helper Cells and Regulatory B Cells Dynamics in Systemic Lupus Erythematosus
Source: PLoS One. 2014 Feb 14;9(2):e88441. doi: 10.1371/journal.pone.0088441 (PMC3925141; doi:10.1371/journal.pone.0088441)

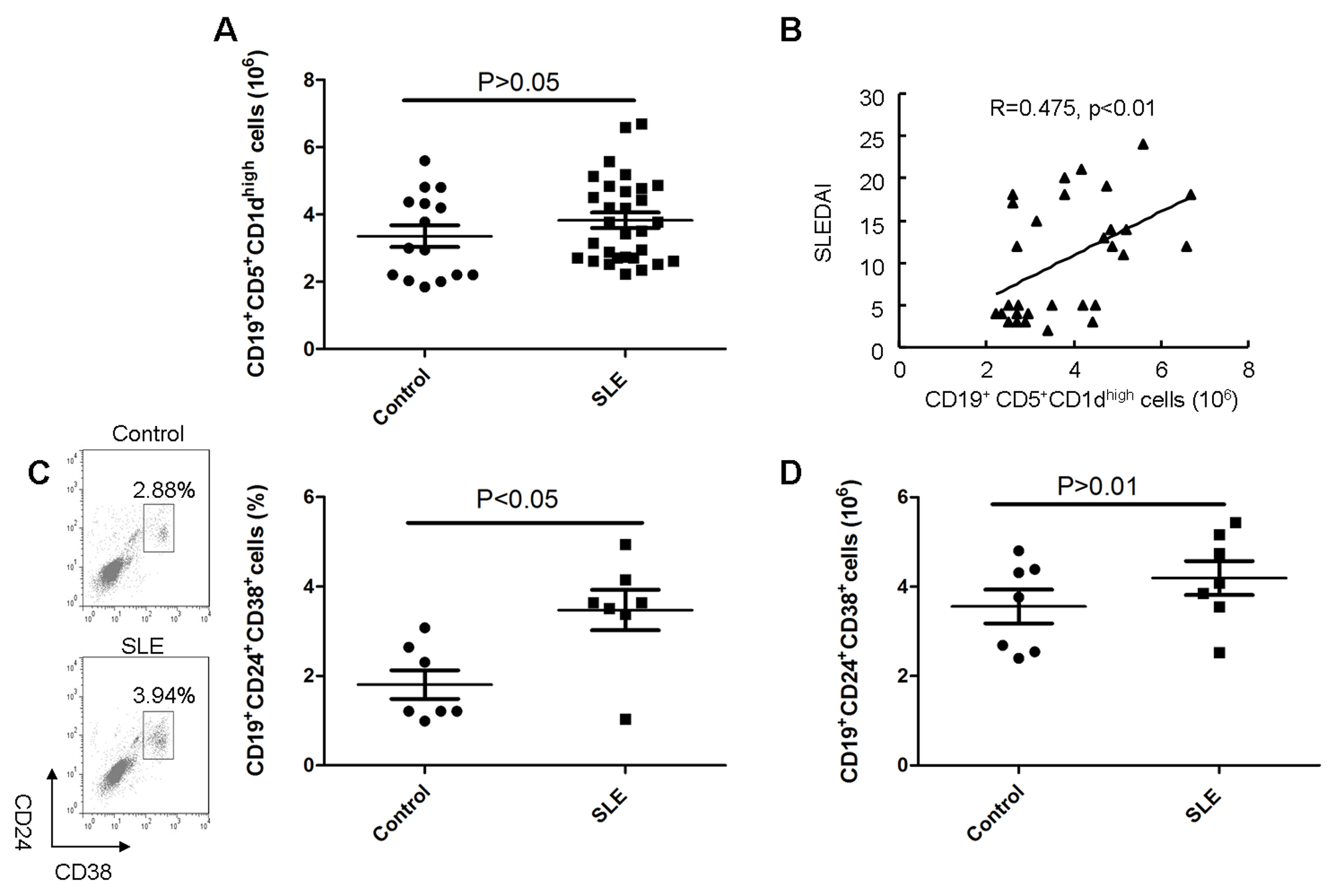

Supplement: Figure S1 — The absolute numbers of Breg cells in SLE patients. (A) The results of flow cytometric analysis of absolute numbers of CD19+CD5+CD1dhigh cells in patients with SLE (n = 30) and healthy controls (n = 15). (B) A positive correlation between the absolute numbers of CD19+ CD5+CD1dhigh cells and the clinical severity of the flare as scored using the SLEDAI (n = 30) was observed. (C) Human PBMCs were labeled with lymphocyte-specific antibodies (CD19, CD24, and CD38). The percentage of CD24+CD38+ cells among a CD19 gate was determined by flow cytometry (left). Results of flow cytometric analysis of percentage of CD24+CD38+ cells among a CD19 gate cells in patients with SLE and control subject (right, n = 7 for each group). (D) The results of flow cytometric analysis of absolute numbers of CD19+CD24+CD38+ cells in patients with SLE and healthy controls (n = 7 for each group). (TIF) [file pone.0088441.s001.tif]

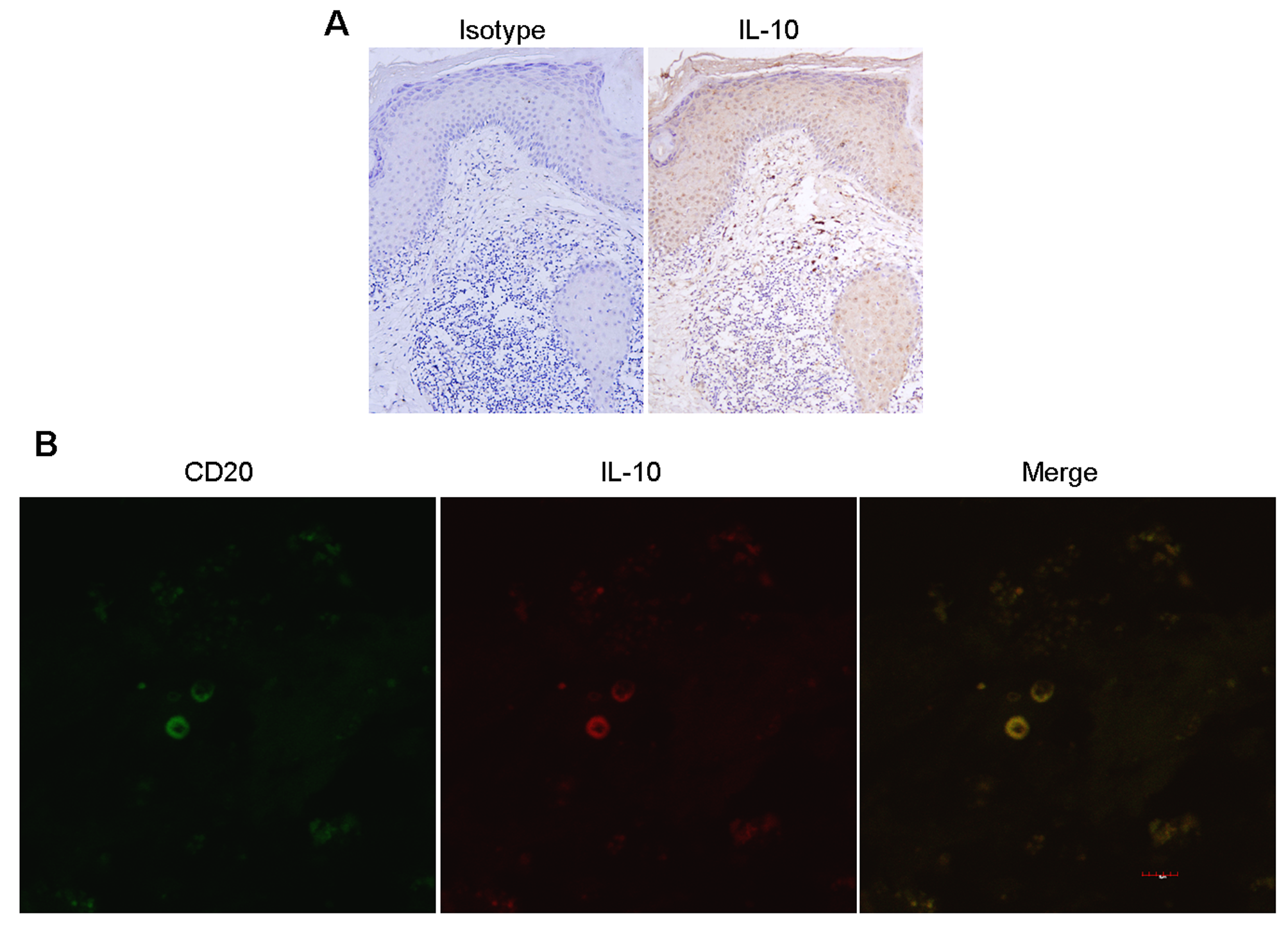

Supplement: Figure S2 — IL-10+ cells in skins of SLE patients. (A) The skin tissues from SLE patient were serially sectioned to obtain consecutive levels. The sections were stained with antibodies to IL-10 and isotype control. (B) The skin tissues from SLE patient were stained with CD20 and IL-10, the CD20+IL-10+ cells were analyzed by immunofluorescence microscopy. (TIF) [file pone.0088441.s002.tif]

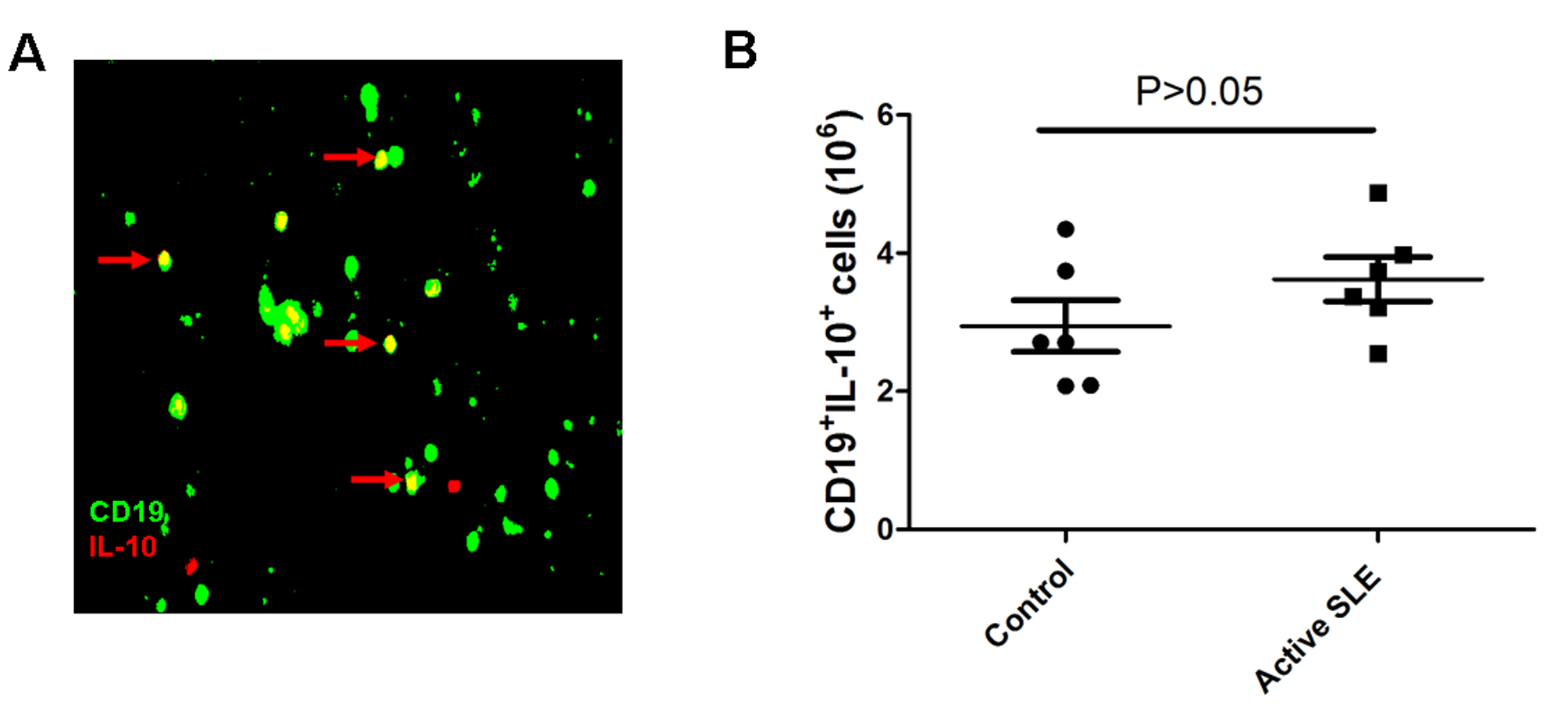

Supplement: Figure S3 — IL-10+ cells in PBMCs of SLE patients. (A) PBMCs were isolated and stimulated with LPS for 24 hours and PIB for the final 5 hours. The presence of CD19+IL-10+ cells in PBMCs from active SLE patients was detected by immunofluorescence microscopy. The arrow indicates typical positive cells. (B) CD19+IL-10+ cells were detected by flow cytometry analysis in a CD19 gate (n = 6 for each group). (TIF) [file pone.0088441.s003.tif]

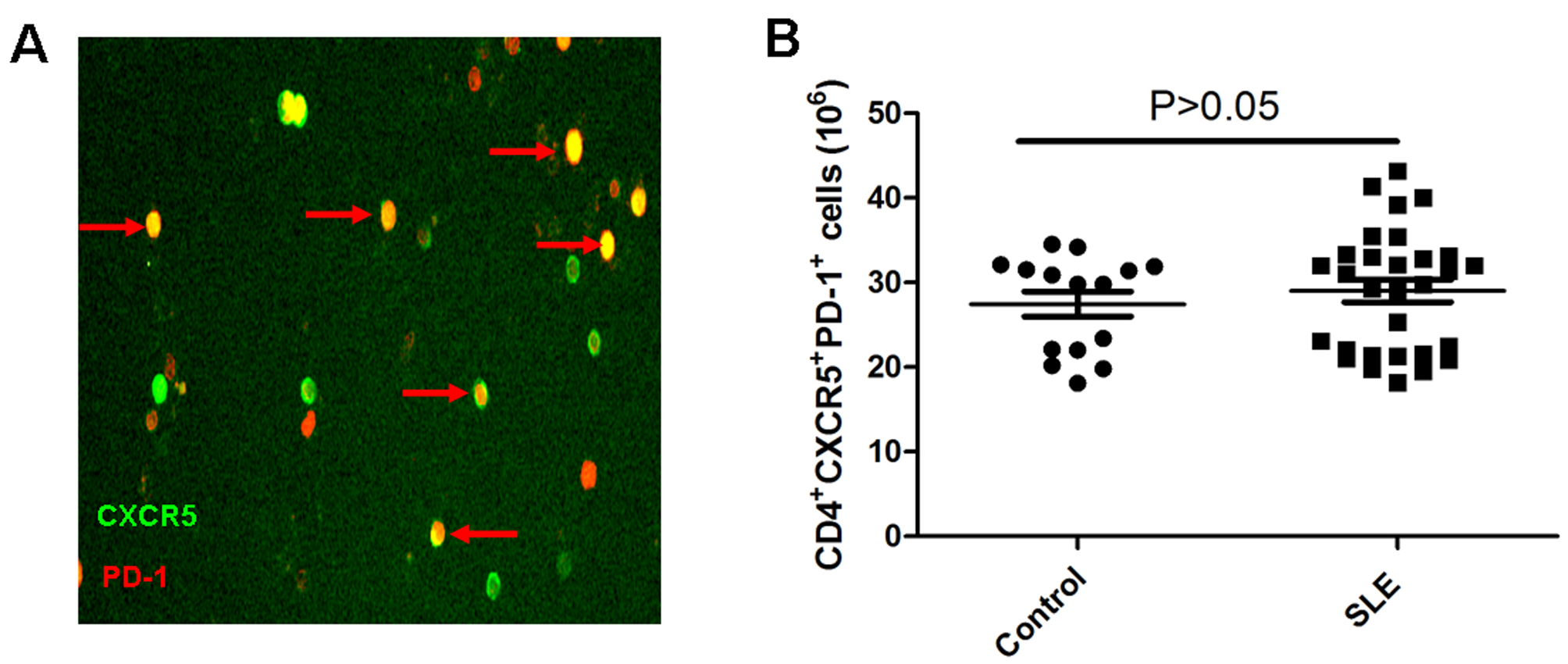

Supplement: Figure S4 — Tfh cells in PBMCs of SLE patients. (A) The presence of CXCR5+PD-1+ cells in PBMCs of active SLE patients was detected by immunofluorescence microscopy. The arrow indicates the typical positive cells. (B) The results of flow cytometric analysis of absolute numbers of CD4+CXCR5+PD-1+ cells in patients with SLE (n = 30) and healthy controls (n = 15). (TIF) [file pone.0088441.s004.tif]
